# Antioxidant Capacities and Total Phenolic Contents of 56 Wild Fruits from South China

**DOI:** 10.3390/molecules15128602

**Published:** 2010-11-29

**Authors:** Li Fu, Bo-Tao Xu, Xiang-Rong Xu, Xin-Sheng Qin, Ren-You Gan, Hua-Bin Li

**Affiliations:** 1 Guangdong Provincial Key Laboratory of Food, Nutrition and Health, School of Public Health, Sun Yat-Sen University, Guangzhou 510080, China; E-Mails: fulilele@hotmail.com (L.F.); ganry_zsu@yahoo.cn (R.G.); 2 Department of Neurosurgery, Nanfang Hospital, Southern Medical University, Guangzhou 510515, China; E-Mail: xbtfl@yahoo.com (B-T.X.); 3 Key Laboratory of Marine Bio-resources Sustainable Utilization, South China Sea Institute of Oceanology, Chinese Academy of Sciences, Guangzhou 510301, China; E-Mail: xuxr@scsio.ac.cn (X-R.X.); 4 College of Forestry, South China Agricultural University, Guangzhou 510640, China; E-Mail: qinxinsheng@scau.edu.cn (X-S.Q.); 5 Liwan District Center for Disease Control and Prevention, Guangzhou 510176, China

**Keywords:** wild fruit, antioxidant capacity, total phenolic content

## Abstract

In order to identify wild fruits possessing high nutraceutical potential, the antioxidant activities of 56 wild fruits from South China were systematically evaluated. The fat-soluble components were extracted with tetrahydrofuran, and the water-soluble ones were extracted with a 50:3.7:46.3 (v/v) methanol-acetic acid-water mixture. The antioxidant capacities of the extracts were evaluated using the ferric reducing antioxidant power (FRAP) and Trolox equivalent antioxidant capacity (TEAC) assays, and their total phenolic contents were measured by the Folin–Ciocalteu method. Most of these wild fruits were analyzed for the first time for their antioxidant activities. Generally, these fruits had high antioxidant capacities and total phenolic contents. A significant correlation between the FRAP value and the TEAC value suggested that antioxidant components in these wild fruits were capable of reducing oxidants and scavenging free radicals. A high correlation between antioxidant capacity and total phenolic content indicated that phenolic compounds could be the main contributors to the measured antioxidant activity. The results showed that fruits of *Eucalyptus robusta*, *Eurya nitida*, *Melastoma sanguineum*, *Melaleuca leucadendron*, *Lagerstroemia indica*, *Caryota mitis*, *Lagerstroemia speciosa* and *Gordonia axillaris* possessed the highest antioxidant capacities and total phenolic contents among those tested, and could be potential rich sources of natural antioxidants and functional foods. The results obtained are very helpful for the full utilization of these wild fruits.

## 1. Introduction

It has been proven that free radicals play an important role in many diseases, such as cardiovascular diseases, cancer, neurodegenerative diseases, diabetes and ageing [1,2,3]. Antioxidants have attracted more and more attention as potential agents for preventing and treating oxidative stress-related diseases. The antioxidants comprise both synthetic and natural antioxidants. The synthetic antioxidants have been widely used in the food industry to prolong the shelf life, but some synthetic antioxidants, such as butylated hydroxytoluene and butylated hydroxyanisole, have been found to be harmful due to their potential toxicity and carcinogenicity [4]. On the other hand, epidemiological studies have found that the intake of fruits and vegetables has a strong inverse correlation with the risk of developing many chronic diseases, such as cardiovascular diseases and cancer [5,6,7,8,9]. Vitamins and polyphenols in fruits and vegetables are considered to be responsible for such antioxidant activity, with polyphenols being the most active [10,11]. The natural antioxidants are expected to be an alternative to synthetic ones because of their potential health benefits [12,13]. Antioxidant activities of many fruits, vegetables, spices, medicinal plants and microalgae have been evaluated, and the results showed that some of them could be rich sources of natural antioxidants [14,15,16,17,18,19,20,21,22,23,24,25], so the search for raw materials containing potent natural antioxidants continues to attract the attention of researchers.

Wild fruits are receiving increasing interest from researchers because of their medicinal properties, nutritional value, vitamin and mineral contents. Concerning their medicinal properties, the most commonly studied benefit is their antioxidant effects [26,27,28,29]. There is an abundance of wild fruits in China because of its vast territory, various landforms and wide-spanning climate. Some of these wild fruits are edible, while some can be used as medicines. However, many wild fruits in China are underutilized, and no report about antioxidant activities of wild fruits from China could be found in the literature.

The aim of this study was to systematically evaluate the antioxidant activities and total phenolic contents of 56 wild fruits from South China, to screen out wild fruits with high antioxidant capacity and total phenolic content, and to investigate the relationship of antioxidant capacity and total phenolic content. The results from this study should prove helpful for the full utilization of these wild fruits.

## 2. Results and Discussion

### 2.1. Antioxidant capacities of the 56 wild fruits

Fifty-six wild fruit samples were collected from different regions of South China, and were authenticated by a botanist. The scientific names of the plants and sampling location of the wild fruit are shown in Table 1. 

**Table 1:** The scientific names, sampling location and FRAP values (μmol Fe(II)/g) of 56 wild fruits.

No.	Scientific Name of Plant	Sampling Location	FRAP values (μmol Fe(II)/g)		
			Water-soluble fraction	Fat-soluble fraction	Total
1	*Mallotus apelta*	Guangdong	6.15 ± 0.36	16.2 ± 1.63	22.4 ± 1.99
2	*Smilax china*	Guangdong	35.0 ± 2.69	84.8 ± 5.86	120 ± 8.55
3	*Gardenia jasminoides*	Guangdong	8.25 ± 0.11	4.42 ± 0.13	12.7 ± 0.24
4	*Helicteres angustifolia*	Guangdong	12.2 ± 0.24	2.15 ± 0.14	14.4 ± 0.38
5	*Eucalyptus robusta*	Guangdong	125 ± 2.49	377 ± 3.92	502 ± 6.41
6	*Ficus benjamina*	Guangdong	6.34 ± 0.38	1.92 ± 0.13	8.26 ± 0.51
7	*Ilex rotunda*	Guangdong	35.9 ± 2.63	19.9 ± 1.26	55.8 ± 3.89
8	*Allamanda schottii*	Guangdong	12.0 ± 1.02	38.4 ± 0.90	50.4 ± 1.92
9	*Areca triandra*	Guangdong	0.67 ± 0.04	0.61 ± 0.04	1.28 ± 0.08
10	*Alpinia zerumbet*	Guangdong	8.87 ± 0.51	6.11 ± 0.34	15.0 ± 0.85
11	*Schefflera heptaphylla*	Guangdong	6.30 ± 0.14	10.6 ± 0.48	16.9 ± 0.62
12	*Ficus hispida*	Guangdong	4.93 ± 0.17	1.33 ± 0.11	6.26 ± 0.28
13	*Melaleuca leucadendron*	Guangdong	68.1 ± 3.48	146 ± 5.38	214 ± 8.86
14	*Lagerstroemia speciosa*	Guangdong	42.2 ± 1.79	182 ± 7.89	225 ± 9.68
15	*Acronychia pedunculata*	Guangdong	4.31 ± 0.26	10.5 ± 0.29	14.8 ± 0.55
16	*Litsea rotundifolia*	Guangdong	17.6 ± 0.80	9.99 ± 0.48	27.6 ± 1.28
17	*Lantana camara*	Guangdong	4.31 ± 0.19	1.81 ± 0.03	6.12 ± 0.22
18	*Dianella ensifolia*	Guangdong	2.66 ± 0.10	3.02 ± 0.18	5.68 ± 0.28
19	*Microcos paniculata*	Guangdong	18.8 ± 0.35	9.71 ± 0.08	28.5 ± 0.43
20	*Melastoma candidum*	Guangdong	95.2 ± 5.89	45.6 ± 2.88	141 ± 8.77
21	*Diplospora dubia*	Guangdong	47.0 ± 2.41	117 ± 6.56	164 ± 8.97
22	*Dolichandrone caudafelina*	Guangdong	41.1 ± 2.39	5.64 ± 0.13	46.7 ± 2.52
23	*Asparagus cochinchinensis*	Hong Kong	1.71 ± 0.06	2.02 ± 0.01	3.73 ± 0.07
24	*Psychotria asiatica*	Hong Kong	41.3 ± 0.11	8.65 ± 0.68	49.9 ± 0.79
25	*Lagerstroemia indica*	Hong Kong	52.8 ± 2.32	98.3 ± 0.32	151 ± 2.64
26	*Nandina domestica*	Hong Kong	51.1 ± 3.38	59.0 ± 0.63	110 ± 4.01
27	*Alpinia hainanensis*	Hong Kong	5.67 ± 0.31	7.66 ± 0.21	13.3 ± 0.52
28	*Gordonia axillaris*	Hong Kong	74.4 ± 3.25	106 ± 7.90	180 ± 11.2
29	*Rhodomyrtus tomentosa*	Hong Kong	45.3 ± 2.77	35.4 ± 1.16	80.7 ± 3.93
30	*Breynia fruticosa*	Hong Kong	8.76 ± 0.68	5.50 ± 0.31	14.3 ± 0.99
31	*Eurya chinensis*	Hong Kong	40.8 ± 2.52	21.6 ± 0.27	62.4 ± 2.79
32	*Duranta erecta*	Hong Kong	5.49 ± 0.40	9.20 ± 0.05	14.7 ± 0.45
33	*Melastoma sanguineum*	Hong Kong	143 ± 2.00	145 ± 8.44	288 ± 10.4
34	*Eurya nitida*	Hong Kong	83.3 ± 2.63	345 ± 9.39	428 ± 12.0
35	*Pyracantha fortuneana*	Hong Kong	18.1 ± 0.14	6.09 ± 0.09	24.2 ± 0.23
36	*Caryota mitis*	Hong Kong	35.6 ± 1.50	138 ± 2.50	174 ± 4.00
37	*Viburnum fordiae*	Jiangxi	75.7 ± 2.06	70.0 ± 1.16	146 ± 3.22
38	*Xanthium sibiricum*	Jiangxi	1.47 ± 0.11	1.70 ± 0.01	3.17 ± 0.12
39	*Celtis sinesis*	Jiangxi	3.17 ± 0.30	1.67 ± 0.08	4.84 ± 0.38
40	*Solanum torvum*	Jiangxi	3.46 ± 0.22	1.78 ± 0.05	5.24 ± 0.27
41	*Melia azedarach*	Jiangxi	8.30 ± 0.39	7.07 ± 0.50	15.4 ± 0.89
42	*Symplocos paniculata*	Jiangxi	6.51 ± 0.19	5.01 ± 0.07	11.5 ± 0.26
43	*Solanum americanum*	Jiangxi	1.32 ± 0.06	2.21 ± 0.11	3.53 ± 0.17
44	*Ardisia crenata*	Jiangxi	13.7 ± 0.60	20.1 ± 0.90	33.9 ± 1.50
45	*Cinnamomum camphora*	Jiangxi	10.2 ± 0.59	10.1 ± 0.80	20.3 ± 1.39
46	*Lagerstroemia indica*	Jiangxi	118 ± 1.49	137 ± 4.26	254 ± 5.75
47	*Parthenocissus dalzielii*	Jiangxi	20.3 ± 1.32	66.5 ± 3.53	86.8 ± 4.85
48	*Loropetalum chinense*	Jiangxi	22.0 ± 0.32	33.2 ± 2.12	55.2 ± 2.44
49	*Rosa laevigata*	Jiangxi	86.9 ± 2.18	58.2 ± 1.13	145 ± 3.31
50	*Viburnum sempervirens*	Jiangxi	35.0 ± 1.01	73.4 ± 4.01	108 ± 5.02
51	*Diospyros kaki*	Jiangxi	1.88 ± 0.13	1.63 ± 0.09	3.51 ± 0.22
52	*Vernici afordii*	Jiangxi	30.6 ± 1.39	51.7 ± 2.10	82.3 ± 3.49
53	*Swida austrosinensis*	Jiangxi	22.0 ± 0.62	35.2 ± 0.54	57.2 ± 1.16
54	*Hibiscus sabdariffa*	Jiangxi	8.68 ± 0.26	7.20 ± 0.29	15.9 ± 0.55
55	*Rhus chinensis*	Jiangxi	21.3 ± 0.30	38.5 ± 0.18	59.8 ± 0.48
56	*Aralia armata*	Jiangxi	1.94 ± 0.04	1.90 ± 0.09	3.84 ± 0.13

The antioxidants in wild fruits are either water-soluble or fat-soluble. When determining total antioxidant activities of wild fruits, both water-soluble and fat-soluble components should be considered. In this study, the fat-soluble components of wild fruits were extracted with tetrahydrofuran, and their water-soluble components were extracted with a mixture of methanol-acetate acid-water [30]. The ferric reducing antioxidant power (FRAP) assay was used to evaluate antioxidant capacities of the 56 wild fruits. The FRAP assay, which is a simple, inexpensive and widely employed method for the evaluation of antioxidant capacity [20], is based on the capacity of antioxidants to reduce ferric(III) ions to ferrous(II) ions [31]. The FRAP values of the 56 wild fruits are given in Table 1. 

As seen from the data in the table, the FRAP values varied from 0.67 ± 0.04 to 143 ± 2.00 μmol Fe(II)/g wet weight with a 213-fold difference for the water-soluble fractions, and eight wild fruits, namely *Melastoma sanguineum* [143 ± 2.00 µmol Fe(II)/g], *Eucalyptus robusta* [125 ± 2.49 µmol Fe(II)/g], *Lagerstroemia indica* [118 ± 1.49 µmol Fe(II)/g], *Melastoma candidum* [95.2 ± 5.89 µmol Fe(II)/g], *Rosa laevigata* [86.9 ± 2.18 µmol Fe(II)/g], *Eurya nitida* [83.3 ± 2.63 µmol Fe(II)/g], *Viburnum fordiae* [75.7 ± 2.06 µmol Fe(II)/g] and *Gordonia axillaris* [74.4 ± 3.25 µmol Fe(II)/g], had the highest antioxidant capacities, while *Areca triandra* had the lowest antioxidant capacity among the 56 wild fruits at 0.67 ± 0.04 μmol Fe(II)/g wet weight. For the fat-soluble fractions, the FRAP values varied from 0.61 ± 0.04 to 377 ± 3.92 μmol Fe(II)/g wet weight with a 618-fold difference, and eight wild fruits, *Eucalyptus robusta* [377 ± 3.92 µmol Fe(II)/g], *Eurya nitida* [345 ± 9.39 µmol Fe(II)/g)], *Lagerstroemia speciosa* [182 ± 7.89 µmol Fe(II)/g], *Melaleuca leucadendron* [146 ± 5.38 µmol Fe(II)/g], *Melastoma sanguineum* [145 ± 8.44 µmol Fe(II)/g)], *Caryota mitis* [138 ± 2.50 µmol Fe(II)/g], *Lagerstroemia indica* [137 ± 4.26 µmol Fe(II)/g] and *Diplospora dubia* [117 ± 6.56 µmol Fe(II)/g], had the highest antioxidant capacities, while *Areca triandra* again had the lowest antioxidant capacity among the 56 tested wild fruits with a value of 0.61 ± 0.04 μmol Fe(II)/g wet weight. For the combined value of water-soluble and fat-soluble fractions, the FRAP values varied from 1.28 ± 0.08 to 502 ± 6.41 μmol Fe(II)/g wet weight with a 392-fold difference, and eight wild fruits, *Eucalyptus robusta* [502 ± 6.41 µmol Fe(II)/g], *Eurya nitida* [428 ± 12.0 µmol Fe(II)/g], *Melastoma sanguineum* [288 ± 10.4 µmol Fe(II)/g], *Lagerstroemia indica* [254 ± 5.75 µmol Fe(II)/g], *Lagerstroemia speciosa* [225 ± 9.68 µmol Fe(II)/g], *Melaleuca leucadendron* [214 ± 8.86 µmol Fe(II)/g], *Gordonia axillaris* [180 ± 11.2 µmol Fe(II)/g] and *Caryota mitis* [174 ± 4.00 µmol Fe(II)/g], had the highest antioxidant capacities.

The antioxidant capacities of plant samples may be influenced by a lot of factors, such as the extraction solvent and test systems used, and cannot be fully described by any one single method. A reliable antioxidant evaluation protocol requires measurement of more than one property because most natural antioxidants are multifunctional, so it is essential to perform different antioxidant activity assessments to take into account the various mechanisms of antioxidant action [32]. Therefore, the Trolox equivalent antioxidant capacity (TEAC) assay was used to evaluate the free radical scavenging capacities of the 56 wild fruits. The TEAC assay is based on the ability of antioxidants to scavenge the ABTS^•+^ radical, and can measure antioxidant capacities of hydrophilic and lipophilic compounds in the same sample [33]. The TEAC assay is a simple, rapid and commonly used method for the evaluation of antioxidant capacity, and could offer reproducible results [21,22]. In the literature, natural and synthetic antioxidants, such as ascorbic acid, vitamin E, butylated hydroxytoluene, butylated hydroxyacetone and Trolox, were often used as positive controls [21,22,34,35]. In this study, Trolox was used, and the results were expressed as µmol Trolox/g wet weight of wild fruit. The TEAC values of the 56 wild fruits are given in Table 2. 

**Table 2:** The TEAC values (μmol Trolox/g) of 56 wild fruits.

No.	Scientific Name of Plant	TEAC values (μmol Trolox/g)		
		Water-soluble fraction	Fat-soluble fraction	Total
1	*Mallotus apelta*	4.06 ± 0.13	22.1 ± 0.82	26.2 ± 0.95
2	*Smilax china*	24.4 ± 1.46	98.2 ± 4.73	123 ± 6.19
3	*Gardenia jasminoides*	2.26 ± 0.04	3.80 ± 0.08	6.06 ± 0.12
4	*Helicteres angustifolia*	5.99 ± 0.29	4.61 ± 0.23	10.6 ± 0.52
5	*Eucalyptus robusta*	184 ± 2.14	957 ± 5.79	1140 ± 7.93
6	*Ficus benjamina*	3.12 ± 0.10	5.78 ± 0.34	8.90 ± 0.44
7	*Ilex rotunda*	19.7 ± 0.13	18.8 ± 0.32	38.5 ± 0.45
8	*Allamanda schottii*	6.75 ± 0.21	30.0 ± 1.73	36.7 ± 1.94
9	*Areca triandra*	0.46 ± 0.02	6.05 ± 0.39	6.51 ± 0.41
10	*Alpinia zerumbet*	6.09 ± 0.28	14.7 ± 0.62	20.8 ± 0.90
11	*Schefflera heptaphylla*	2.02 ± 0.14	4.25 ± 0.11	6.27 ± 0.25
12	*Ficus hispida*	2.35 ± 0.18	2.83 ± 0.06	5.18 ± 0.24
13	*Melaleuca leucadendron*	138 ± 4.71	323 ± 2.17	461 ± 6.88
14	*Lagerstroemia speciosa*	58.0 ± 0.59	264 ± 16.8	322 ± 17.4
15	*Acronychia pedunculata*	2.73 ± 0.08	19.2 ± 0.05	21.9 ± 0.13
16	*Litsea rotundifolia*	11.5 ± 0.56	16.8 ± 0.65	28.3 ± 1.21
17	*Lantana camara*	1.75 ± 0.09	3.68 ± 0.17	5.43 ± 0.26
18	*Dianella ensifolia*	0.62 ± 0.03	3.70 ± 0.04	4.32 ± 0.07
19	*Microcos paniculata*	9.61 ± 0.16	15.2 ± 0.05	24.8 ± 0.21
20	*Melastoma candidum*	93.8 ± 4.47	88.6 ± 1.00	182 ± 5.47
21	*Diplospora dubia*	66.4 ± 0.26	91.0 ± 0.80	157 ± 1.06
22	*Dolichandrone caudafelina*	17.3 ± 0.56	17.6 ± 0.02	34.9 ± 0.58
23	*Asparagus cochinchinensis*	0.37 ± 0.01	3.68 ± 0.19	4.05 ± 0.20
24	*Psychotria asiatica*	36.1 ± 1.58	33.6 ± 1.36	69.7 ± 2.94
25	*Lagerstroemia indica*	73.2 ± 0.81	119 ± 1.12	192 ± 1.93
26	*Nandina domestica*	20.7 ± 0.24	57.3 ± 0.54	78.0 ± 0.78
27	*Alpinia hainanensis*	3.73 ± 0.15	26.8 ± 1.63	30.5 ± 1.78
28	*Gordonia axillaris*	79.4 ± 0.40	265 ± 11.7	344 ± 12.1
29	*Rhodomyrtus tomentosa*	29.7 ± 0.94	48.7 ± 1.93	78.4 ± 2.87
30	*Breynia fruticosa*	4.83 ± 0.12	11.0 ± 0.42	15.8 ± 0.54
31	*Eurya chinensis*	33.5 ± 0.23	40.7 ± 0.78	74.2 ± 1.01
32	*Duranta erecta*	1.76 ± 0.08	19.0 ± 0.88	20.8 ± 0.96
33	*Melastoma sanguineum*	130 ± 8.77	275 ± 12.7	404 ± 21.5
34	*Eurya nitida*	56.0 ± 2.52	422 ± 11.6	478 ± 14.1
35	*Pyracantha fortuneana*	7.40 ± 0.25	18.1 ± 0.88	25.5 ± 1.13
36	*Caryota mitis*	27.3 ± 0.35	361 ± 10.4	389 ± 10.8
37	*Viburnum fordiae*	88.0 ± 2.32	54.8 ± 3.90	143 ± 6.22
38	*Xanthium sibiricum*	12.0 ± 0.01	11.4 ± 0.14	23.3 ± 0.15
39	*Celtis sinesis*	11.3 ± 0.08	12.3 ± 0.11	23.6 ± 0.19
40	*Solanum torvum*	11.3 ± 0.05	10.6 ± 0.16	22.0 ± 0.21
41	*Melia azedarach*	10.5 ± 0.10	7.03 ± 0.25	17.5 ± 0.35
42	*Symplocos paniculata*	10.4 ± 0.10	14.0 ± 0.52	24.4 ± 0.62
43	*Solanum americanum*	12.2 ± 0.03	10.9 ± 0.15	23.1 ± 0.18
44	*Ardisia crenata*	5.91 ± 0.15	39.2 ± 2.06	45.1 ± 2.21
45	*Cinnamomum camphora*	9.65 ± 0.11	8.14 ± 0.09	17.8 ± 0.20
46	*Lagerstroemia indica*	64.1 ± 3.26	45.0 ± 1.87	109 ± 5.13
47	*Parthenocissus dalzielii*	19.6 ± 1.76	88.6 ± 3.08	108 ± 4.84
48	*Loropetalum chinense*	16.5 ± 1.40	46.1 ± 0.56	62.6 ± 1.96
49	*Rosa laevigata*	67.7 ± 0.32	78.4 ± 4.51	146 ± 4.83
50	*Viburnum sempervirens*	17.8 ± 0.58	96.4 ± 1.56	114 ± 2.14
51	*Diospyros kaki*	0.77 ± 0.03	2.95 ± 0.17	3.72 ± 0.20
52	*Vernici afordii*	18.2 ± 0.43	92.4 ± 3.67	111 ± 4.10
53	*Swida austrosinensis*	14.5 ± 0.20	40.0 ± 2.72	54.5 ± 2.92
54	*Hibiscus sabdariffa*	4.34 ± 0.16	9.50 ± 0.23	13.8 ± 0.39
55	*Rhus chinensis*	29.0 ± 1.60	86.4 ± 5.28	115 ± 6.88
56	*Aralia armata*	0.91 ± 0.03	2.47 ± 0.12	3.38 ± 0.15

As seen from Table 2, the TEAC values varied from 0.37 ± 0.01 to 184 ± 2.14 μmol Trolox/g wet weight with a 496-fold difference for the water-soluble fractions, and eight wild fruits, *Eucalyptus robusta* [184 ± 2.14 μmol Trolox/g], *Melaleuca leucadendron* [138 ± 4.71 μmol Trolox/g], *Melastoma sanguineum* [130 ± 8.77 μmol Trolox/g], *Melastoma candidum* [93.8 ± 4.47 μmol Trolox/g], *Viburnum fordiae* [88.0 ± 2.32 μmol Trolox/g], *Gordonia axillaris* [79.4 ± 0.40 μmol Trolox/g], *Lagerstroemia indica* [73.2 ± 0.81 μmol Trolox/g] and *Rosa laevigata* [67.7 ± 0.32 μmol Trolox/g], had the highest free radical scavenging capacities, while in this assay *Asparagus cochinchinensis* with 0.37 ± 0.01 μmol Trolox/g wet weight showed the lowest free radical scavenging capacity among the tested wild fruits. For the fat-soluble fractions, the TEAC values varied from 2.47 ± 0.12 to 957 ± 5.79 μmol Trolox/g wet weight with a 387–fold difference, and eight wild fruits, *Eucalyptus robusta* [957 ± 5.79 μmol Trolox/g], *Eurya nitida* [422 ± 11.6 μmol Trolox/g], *Caryota mitis* [361 ± 10.4 μmol Trolox/g], *Melaleuca leucadendron* [323 ± 2.17 μmol Trolox/g], *Melastoma sanguineum* [275 ± 12.7 μmol Trolox/g], *Gordonia axillaris* [265 ± 11.7 μmol Trolox/g], *Lagerstroemia speciosa* [264 ± 16.8 μmol Trolox/g] and *Lagerstroemia indica* [119 ± 1.12 μmol Trolox/g], had the highest free radical scavenging capacities, whereas *Aralia armata* showed the lowest free radical scavenging capacity with 2.47 ± 0.12 μmol Trolox/g wet weight. For the combined value of the water-soluble and fat-soluble fractions, the TEAC values varied from 3.38 ± 0.15 to 1,140 ± 7.93 μmol Trolox/g wet weight with a 337–fold difference, and eight wild fruits, *Eucalyptus robusta* [1,140 ± 7.93 μmol Trolox/g], *Eurya nitida* [478 ± 14.1 μmol Trolox/g], *Melaleuca leucadendron* [461 ± 6.88 μmol Trolox/g], *Melastoma sanguineum* [404 ± 21.5 μmol Trolox/g], *Caryota mitis* [389 ± 10.8 μmol Trolox/g], *Gordonia axillaris* [344 ± 12.1 μmol Trolox/g], *Lagerstroemia speciosa* [322 ± 17.4 μmol Trolox/g] and *Lagerstroemia indica* [192 ± 1.93 μmol Trolox/g], had the highest free radical scavenging capacities.

Eight wild fruits, *Eucalyptus robusta*, *Eurya nitida*, *Melastoma sanguineum*, *Melaleuca leucadendron*, *Lagerstroemia indica*, *Caryota mitis*, *Lagerstroemia speciosa* and *Gordonia axillaris*, had the highest antioxidant activities among the 56 tested wild fruits based on the consideration of the combined results obtained by FRAP and TEAC assays. These wild fruits exhibited quite high antioxidant activities when compared with some vegetables and common edible fruits [21]. Their antioxidant capacities were also higher than those of other wild fruits reported in the literature [36]. Because of their high antioxidant activities, it could be speculated that these wild fruits could be beneficial for the diseases caused by oxidative stress, and might be developed into functional foods or drugs in the future.

**Figure 1:**
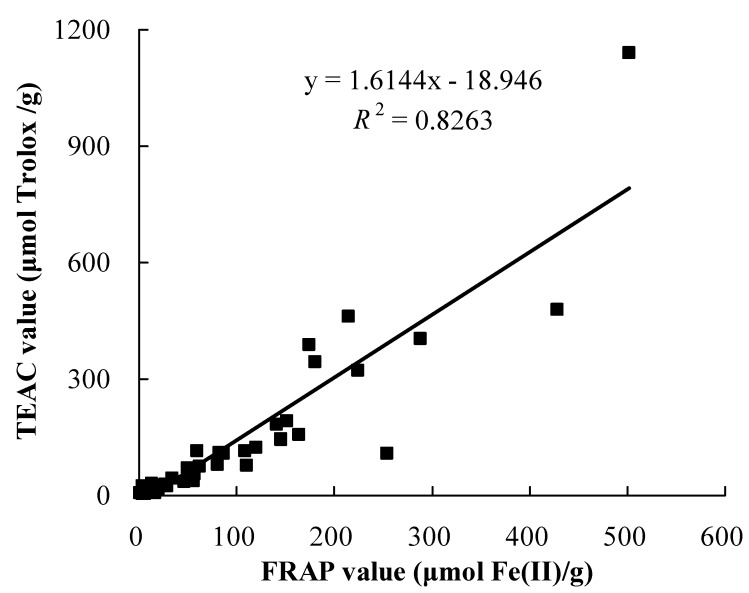
Correlation between total antioxidant capacities measured by the FRAP and TEAC assays.

The correlation between total antioxidant capacities obtained from FRAP and TEAC assays are shown in Figure 1. The results exhibited a positive linear correlation (*R*^2^ = 0.8263) between them. In addition, the results from water-soluble and fat-soluble fractions also showed a positive linear correlation between FRAP and TEAC values (Table 3, *R*^2^ = 0.7895 and 0.8334, respectively). These results suggested that antioxidant components in these wild fruits could reduce oxidants (such as ferric ions) and scavenge free radicals.

**Table 3:** The relation of several evaluation parameters.

Parameter	Water-soluble fraction	Fat-soluble fraction	Total
TEAC value *vs*	Y = 1.0038X − 2.1363	Y = 1.8305X − 8.6945	Y = 1.6144X − 18.946
FRAP value	*R*^2^ = 0.7895	*R*^2^ = 0.8334	*R*^2^ = 0.8263
FRAP value *vs*	Y= 10.063X + 0.9349	Y = 9.6777X − 12.752	Y = 9.6354X − 10.3
Total phenolic content	*R*^2^ = 0.7956	*R*^2^ = 0.9051	*R*^2^ = 0.8916
TEAC value *vs*	Y = 11.978X − 6.6924	Y = 18.997X − 40.128	Y = 16.995X − 48.868
Total phenolic content	*R*^2^ = 0.8834	*R*^2^ = 0.8675	*R*^2^ = 0.8793

### 2.2. Total phenolic content of 56 wild fruits

The total phenolic contents of 56 wild fruits were estimated using the Folin–Ciocalteu method, which relies on the transfer of electrons from phenolic compounds to the Folin–Ciocalteu reagent in alkaline medium, and is a simple, rapid and reproducible method [22,37]. As seen from Table 4, the total phenolic contents varied from 0.06 ± 0.01 to 14.1 ± 0.56 mg GAE/g wet weight with a 235-fold difference for the water-soluble fractions, and eight wild fruits, *Eucalyptus robusta* [14.1 ± 0.56 mg GAE/g], *Melastoma sanguineum* [9.68 ± 0.10 mg GAE/g], *Melaleuca leucadendron* [9.28 ± 0.37 mg GAE/g], *Melastoma candidum* [8.56 ± 0.30 mg GAE/g], *Gordonia axillaris* [7.88 ± 0.44 mg GAE/g], *Diplospora dubia* [7.49 ± 0.42 mg GAE/g], *Rosa laevigata* [7.45 ± 0.28 mg GAE/g] and *Eurya* nitida [6.27 ± 0.36 mg GAE/g] showed the highest total phenolic contents, while *Areca triandra* had the lowest total phenolic content with 0.06 ± 0.01 mg GAE/g wet weight. For the fat-soluble fractions, the total phenolic contents varied from 0.38 ± 0.02 to 40.7 ± 2.49 mg GAE/g wet weight with a 107–fold difference, and eight wild fruits, *Eucalyptus robusta* [40.7 ± 2.49 mg GAE/g], *Eurya nitida* [28.8 ± 0.60 mg GAE/g], *Caryota mitis* [17.5 ± 0.71 mg GAE/g], *Gordonia axillaris* [16.7 ± 0.64 mg GAE/g], *Melaleuca leucadendron* [16.4 ± 1.22 mg GAE/g], *Lagerstroemia speciosa* [14.9 ± 0.32 mg GAE/g], *Viburnum sempervirens* [13.9 ± 0.12 mg GAE/g] and *Melastoma sanguineum* [13.6 ± 0.39 mg GAE/g], showed the highest total phenolic contents. Meanwhile, Diospyros kaki had the lowest total phenolic content with 0.38 ± 0.02 mg GAE/g wet weight. For the combined value of the water-soluble and fat-soluble fractions, the total phenolic contents varied from 0.49 ± 0.04 to 54.8 ± 3.05 mg GAE/g wet weight with a 112–fold difference, and eight wild fruits, *Eucalyptus robusta* [54.8 ± 3.05 mg GAE/g], *Eurya nitida* [35.0 ± 0.96 mg GAE/g], *Melaleuca leucadendron* [25.6 ± 1.59 mg GAE/g], *Gordonia axillaris* [24.6 ± 1.08 mg GAE/g], *Melastoma sanguineum* [23.3 ± 0.49 mg GAE/g], *Caryota mitis* [21.4 ± 0.85 mg GAE/g], *Lagerstroemia speciosa* [20.1 ± 0.35 mg GAE/g] and *Viburnum sempervirens* [18.5 ± 0.40 mg GAE/g], showed the highest total phenolic contents. The total phenolic contents of these eight wild fruits were very high when compared with other vegetables and common edible fruits reported in the literature [21]. Their total phenolic contents were also higher than those of wild fruits reported in the literature [36]. In addition, it should be pointed out that some non-phenolic reducing compounds, such as organic acids and sugars, could interfere the determination of total phenolic contents by the Folin–Ciocalteu method, which leads to an overvaluation of the phenolic content. Furthermore, different phenolics might present different responses with the Folin Ciocalteu reagent, such as gallic acid and rutin have similar behaviours, but several flavonoids present low absorption, which leads to an underestimation of various compounds [38,39,40,41]. 

**Table 4:** The total phenolic contents (mg GAE/g) of 56 wild fruits.

No.	Scientific Name of Plant	Total phenolic contents (mg GAE/g)		
		Water-soluble fraction	Fat-soluble fraction	Total
1	*Mallotus apelta*	1.74 ± 0.10	6.11 ± 0.18	7.85 ± 0.28
2	*Smilax china*	4.80 ± 0.22	11.0 ± 0.56	15.8 ± 0.78
3	*Gardenia jasminoides*	0.72 ± 0.01	1.16 ± 0.06	1.88 ± 0.07
4	*Helicteres angustifolia*	1.07 ± 0.04	1.05 ± 0.06	2.12 ± 0.10
5	*Eucalyptus robusta *	14.1 ± 0.56	40.7 ± 2.49	54.8 ± 3.05
6	*Ficus benjamina*	0.68 ± 0.02	0.95 ± 0.05	1.63 ± 0.07
7	*Ilex rotunda*	2.96 ± 0.06	3.36 ± 0.28	6.32 ± 0.34
8	*Allamanda schottii*	1.31 ± 0.04	7.37 ± 0.44	8.68 ± 0.48
9	*Areca triandra*	0.06 ± 0.01	0.43 ± 0.03	0.49 ± 0.04
10	*Alpinia zerumbet*	1.29 ± 0.10	2.91 ± 0.21	4.20 ± 0.31
11	*Schefflera heptaphylla*	0.54 ± 0.03	1.31 ± 0.06	1.85 ± 0.09
12	*Ficus hispida*	0.51 ± 0.02	0.75 ± 0.02	1.26 ± 0.04
13	*Melaleuca leucadendron*	9.28 ± 0.37	16.4 ± 1.22	25.6 ± 1.59
14	*Lagerstroemia speciosa*	5.22 ± 0.03	14.9 ± 0.32	20.1 ± 0.35
15	*Acronychia pedunculata*	0.46 ± 0.01	3.13 ± 0.04	3.59 ± 0.05
16	*Litsea rotundifolia*	2.06 ± 0.12	3.79 ± 0.30	5.85 ± 0.42
17	*Lantana camara*	0.62 ± 0.02	0.92 ± 0.01	1.54 ± 0.03
18	*Dianella ensifolia*	0.26 ± 0.02	1.15 ± 0.02	1.41 ± 0.04
19	*Microcos paniculata*	2.68 ± 0.06	3.60 ± 0.13	6.28 ± 0.19
20	*Melastoma candidum*	8.56 ± 0.30	7.61 ± 0.68	16.2 ± 0.98
21	*Diplospora dubia*	7.49 ± 0.42	10.9 ± 0.12	18.4 ± 0.54
22	*Dolichandrone caudafelina*	3.15 ± 0.20	2.48 ± 0.02	5.63 ± 0.22
23	*Asparagus cochinchinensis*	0.29 ± 0.01	0.61 ± 0.03	0.90 ± 0.04
24	*Psychotria asiatica*	3.55 ± 0.24	3.73 ± 0.15	7.28 ± 0.39
25	*Lagerstroemia indica*	5.42 ± 0.17	12.0 ± 0.07	17.4 ± 0.24
26	*Nandina domestica*	4.25 ± 0.20	7.72 ± 0.39	12.0 ± 0.59
27	*Alpinia hainanensis*	0.78 ± 0.02	2.00 ± 0.09	2.78 ± 0.11
28	*Gordonia axillaris*	7.88 ± 0.44	16.7 ± 0.64	24.6 ± 1.08
29	*Rhodomyrtus tomentosa*	2.71 ± 0.07	4.07 ± 0.13	6.78 ± 0.20
30	*Breynia fruticosa*	1.22 ± 0.04	1.83 ± 0.07	3.05 ± 0.11
31	*Eurya chinensis*	3.42 ± 0.05	3.85 ± 0.06	7.27 ± 0.11
32	*Duranta erecta*	0.43 ± 0.02	1.80 ± 0.02	2.23 ± 0.04
33	*Melastoma sanguineum*	9.68 ± 0.10	13.6 ± 0.39	23.3 ± 0.49
34	*Eurya nitida*	6.27 ± 0.36	28.8 ± 0.60	35.0 ± 0.96
35	*Pyracantha fortuneana*	1.31 ± 0.04	2.03 ± 0.07	3.34 ± 0.11
36	*Caryota mitis*	3.87 ± 0.14	17.5 ± 0.71	21.4 ± 0.85
37	*Viburnum fordiae*	5.60 ± 0.44	10.6 ± 0.20	16.2 ± 0.64
38	*Xanthium sibiricum*	0.20 ± 0.01	0.45 ± 0.02	0.65 ± 0.03
39	*Celtis sinesis*	0.42 ± 0.02	0.65 ± 0.03	1.07 ± 0.05
40	*Solanum torvum*	0.48 ± 0.01	0.49 ± 0.04	0.97 ± 0.05
41	*Melia azedarach*	0.76 ± 0.03	1.44 ± 0.05	2.20 ± 0.08
42	*Symplocos paniculata*	0.57 ± 0.03	1.38 ± 0.01	1.95 ± 0.04
43	*Solanum americanum*	0.16 ± 0.01	0.47 ± 0.01	0.63 ± 0.02
44	*Ardisia crenata*	0.85 ± 0.03	2.57 ± 0.13	3.42 ± 0.16
45	*Cinnamomum camphora*	0.92 ± 0.04	2.28 ± 0.09	3.20 ± 0.13
46	*Lagerstroemia indica*	4.08 ± 0.02	8.01 ± 0.28	12.1 ± 0.30
47	*Parthenocissus dalzielii*	3.93 ± 0.27	11.8 ± 0.63	15.8 ± 0.90
48	*Loropetalum chinense*	2.43 ± 0.12	5.08 ± 0.31	7.51 ± 0.43
49	*Rosa laevigata*	7.45 ± 0.28	7.34 ± 0.21	14.8 ± 0.49
50	*Viburnum sempervirens*	4.62 ± 0.28	13.9 ± 0.12	18.5 ± 0.40
51	*Diospyros kaki*	0.15 ± 0.01	0.38 ± 0.02	0.53 ± 0.03
52	*Vernici afordii*	2.98 ± 0.15	9.24 ± 0.04	12.2 ± 0.19
53	*Swida austrosinensis*	2.09 ± 0.16	4.81 ± 0.24	6.90 ± 0.40
54	*Hibiscus sabdariffa*	0.60 ± 0.01	1.45 ± 0.05	2.05 ± 0.06
55	*Rhus chinensis*	4.74 ± 0.42	12.4 ± 1.07	17.2 ± 1.49
56	*Aralia armata*	0.24 ± 0.01	0.71 ± 0.01	0.95 ± 0.02

### 2.3. Correlation between antioxidant capacities and total phenolic content

**Figure 2:**
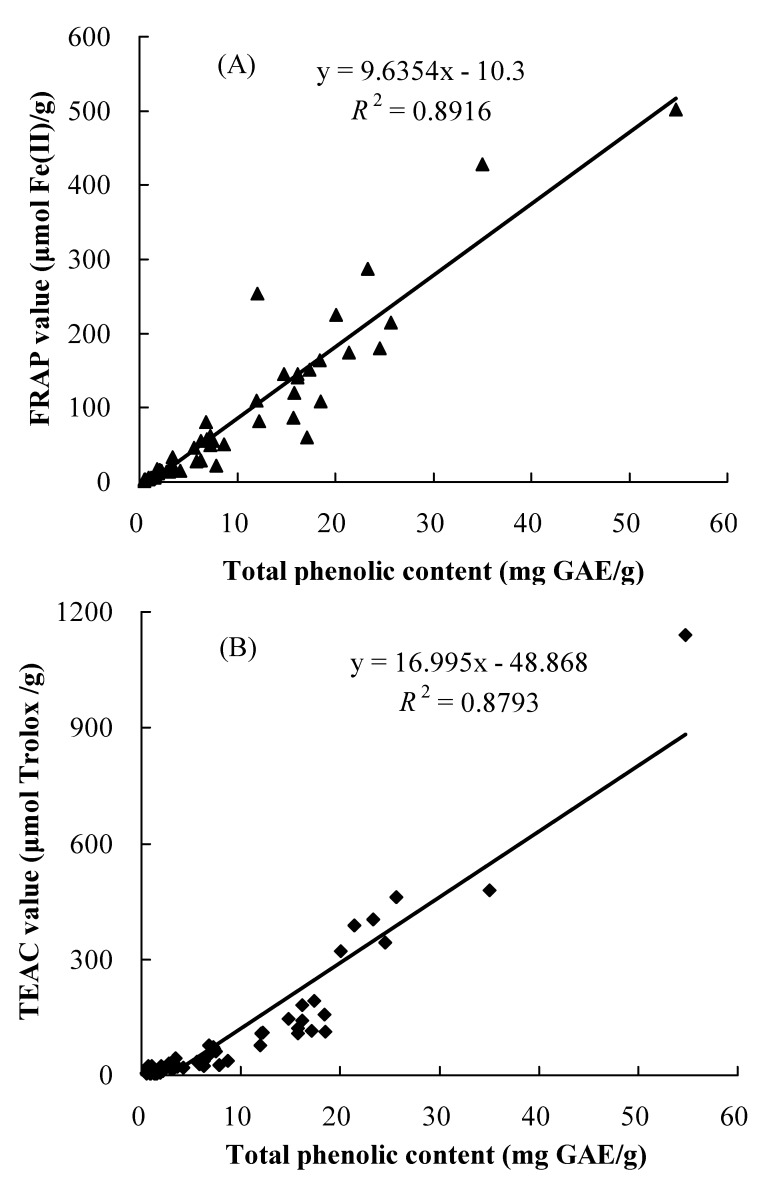
Correlation between the antioxidant capacities and total phenolic content. Antioxidant capacities were measured by the FRAP assay (A) and TEAC assay (B), respectively. GAE: gallic acid equivalents.

The correlation between total antioxidant capacities and the total phenolic content of the 56 tested wild fruits is shown in Figure 2. The results showed a positive linear correlation between the antioxidant capacities and total phenolic content (*R*^2^ = 0.8916 and 0.8793 for the results from the FRAP and TEAC assays, respectively). In addition, the results from the water-soluble fractions showed a positive linear correlation between the antioxidant capacity and total phenolic content (Table 3, *R*^2^ = 0.7956 and 0.8834 for the results from the FRAP and TEAC assays, respectively). Furthermore, the results from the fat-soluble fractions also showed a positive linear correlation between the antioxidant capacity and total phenolic content (Table 3, *R*^2^ = 0.9051 and 0.8675 for the results from the FRAP and TEAC assays, respectively). These results indicated that the phenolic compounds could be the main contributor of the antioxidant activities of these wild fruits. This result was in agreement with many previous studies [26,27].

Comparison of FRAP value, TEAC value and total phenolic content between water-soluble fraction and fat-soluble fraction showed that there was a significant difference in TEAC values (*P* = 0.010 < 0.05) and total phenolic contents (*P* = 0.005 < 0.05), but no significant difference in FRAP values (*P* = 0.954 > 0.05). The fat-soluble fractions possessed stronger scavenging free radical activity and higher phenolic content than the water-soluble fractions. In addition, comparison of FRAP value, TEAC value and total phenolic content of the wild fruits among different sampling locations: Guangdong province, Hong Kong and Jiangxi province, showed that there was no significant difference in FRAP values (*P* = 0.257 > 0.05), TEAC values (*P* = 0.200 > 0.05) and total phenolic contents (*P* = 0.226 > 0.05). Furthermore, three wild fruits, *Gordonia axillaris*, *Rhodomyrtus tomentosa* and *Caryota mitis*, were simultaneously collected both in Guangdong province and Hong Kong. Although their FRAP value, TEAC value and total phenolic content were different (data not shown), there was no significant difference in the three parameters. Although these wild fruits grow in different regions of South China, three regions all belong to the sub-tropical climate.

## 3. Experimental

### 3.1. Chemicals and wild fruits

Folin–Ciocalteu’s phenol reagent, 2,4,6-tri(2-pyridyl)-*s*-triazine (TPTZ), 6-hydroxy-2,5,7,8-tetramethylchromane-2-carboxylic acid (Trolox), 2,2'-azinobis(3-ethylbenothiazoline-6-sulfonic acid) diammonium salt (ABTS) and gallic acid were purchased from Sigma–Aldrich (St. Louis, MO, USA). Iron (III) chloride hexahydrate, iron (II) sulfate heptahydrate, acetic acid, sodium acetate, potassium persulphate, tetrahydrofuran and sodium carbonate were obtained from Tianjing Chemical Factory (Tianjing, China). Ethanol, methanol and hydrochloric acid were obtained from Kelong Chemical Factory (Chengdu, China). All chemicals used in the experiments were of analytical grade, and deionized water was used throughout. Fifty-six wild fruit samples were collected from various locations in South China, and are authenticated by the botanist Dr. Xin-Sheng Qin from the College of Forestry, South China Agricultural University. The scientific names of the plants and sampling sites of the wild fruit are shown in Table 1. 

### 3.2. Sample preparation

The wild fruits were washed with deionized water to remove dirt on their peels, and were given an airing at room temperature. Then, the wild fruits were ground into fine particles/slurry with a special grinder for herbal medicines. A weighed amount (about 1.00 g) of these particles was extracted with tetrahydrofuran (10 mL) at room temperature for 30 min in a shaking water bath according to the literature [30]. The sample was centrifuged at 3,500 rpm for 30 min, and the supernatant was collected. The residue was extracted with the same solvent twice, and the supernatants were combined, which would be used for the evaluation of antioxidant capacity and total phenolic content. Subsequently, the residue was extracted with a mixture of methanol-acetic acid-water (10 mL, 50:3.7:46.3, v/v). The extraction procedure was repeated twice, and the supernatants were combined for the evaluation of antioxidant capacity and total phenolic content.

### 3.3. Ferric-reducing antioxidant power (FRAP) assay

The FRAP assay was carried out according to the procedure described in the literature with minor modifications [31,42]. Briefly, the FRAP reagent was prepared from sodium acetate buffer (300 mM, pH 3.6), 10 mM TPTZ solution (40 mM HCl as solvent) and 20 mM iron (III) chloride solution in a volume ratio of 10:1:1, respectively. The FRAP reagent was prepared freshly daily and warmed to 37 °C in a water bath before use. One hundred microliters of the diluted sample was added to a portion of the FRAP reagent (3 mL). After 4 min, the absorbance of the reaction mixture was measured at 593 nm using a Shimadzu UV-2450 ultraviolet-visible spectrophotometer (Japan). The standard curve was constructed using FeSO_4_ solution, and the results were expressed as µmol Fe(II)/g wet weight of wild fruit.

### 3.4. Trolox equivalent antioxidant capacity (TEAC) assay

The TEAC assay was carried out according to the method established in the literature with slight modifications [33]. Briefly, the ABTS^•+^ stock solution was prepared from 7 mM ABTS and 2.45 mM potassium persulfate in a volume ratio of 1:1, and then incubated in the dark for 16 h at room temperature and used within 2 days. The ABTS^•+^ working solution was prepared by diluting the stock solution with ethanol to an absorbance of 0.70 ± 0.05 at 734 nm. All samples were diluted approximately to provide 20–80% inhibition of the blank absorbance. One hundred microliters of the diluted sample was mixed with ABTS^•+^ working solution (3.8 mL) and after 6 min of incubation at room temperature, the absorbance of the reaction mixture was measured at 734 nm, and the percent of inhibition of absorbance at 734 nm was calculated. Trolox was used as a reference standard, and the results were expressed as µmol Trolox/g wet weight of wild fruit.

### 3.5. Determination of total phenolic content

Total phenolic contents were determined according to the literature [22,37]. Briefly, the diluted sample (0.50 mL) was added to 1:10 diluted Folin–Ciocalteu reagent (2.5 mL). After 4 min, saturated sodium carbonate solution (about 75 g/L, 2 mL) was added. The absorbance of the reaction mixture was measured at 760 nm after incubation for 2 h at room temperature. Gallic acid was used as a reference standard, and the results were expressed as milligram gallic acid equivalent (mg GAE)/g wet weight of wild fruit.

### 3.6. Statistical analysis

All the experiments were performed in triplicate, and the results were expressed as mean ± SD (standard deviation). Statistical analysis was performed using SPSS 13.0 and Excel 2003. The difference was considered significant at *p* < 0.05.

## 4. Conclusions

The antioxidant activities and total phenolic contents of 56 wild fruits collected from South China were evaluated. Generally, these wild fruits had high antioxidant capacities and total phenolic contents. A significant correlation between the FRAP value and the TEAC value suggested that antioxidant components in these wild fruits were capable of reducing oxidants and scavenging free radicals. A high correlation between antioxidant capacity and total phenolic content indicated that phenolic compounds could be the main contributors to the antioxidant activity of these wild fruits. The fruits of *Eucalyptus robusta*, *Eurya nitida*, *Melastoma sanguineum*, *Melaleuca leucadendron*, *Lagerstroemia indica*, *Caryota mitis*, *Lagerstroemia speciosa* and *Gordonia axillaris* possessed the highest antioxidant capacities and total phenolic contents among the wild fruits tested. These fruits could be potential rich resources of natural antioxidants, and could be developed into functional foods or drug for the prevention and treatment of diseases caused by oxidative stress. In the future, the specific components with high antioxidant capacities in these wild fruits should be isolated and identified, and explored for their health effects against oxidative stress.
